# The effect of salience of rewards on effort-based decision making in psychotic disorders

**DOI:** 10.1186/s12888-022-04274-7

**Published:** 2022-10-13

**Authors:** Katharina E. Renz, Tania M. Lincoln

**Affiliations:** Clinical Psychology and Psychotherapy, Institute of Psychology, Faculty of Psychology and Human Movement Science, Universität Hamburg, Von-Melle-Park 5, 20146 Hamburg, Germany

**Keywords:** Amotivation, Negative symptoms, schizophrenia, Balloon effort task, Intervention

## Abstract

**Background:**

Although motivational negative symptoms account for reduced functioning and quality of life in individuals with psychotic disorders, the underlying mechanisms are yet not fully understood. Neuroimaging studies suggest that an impaired perception of reward cues could result in a lack of incentive value that then leads to a decrease in goal-directed behavior. Therefore, the aim of this study was to test the effect of increasing the salience of reward cues on goal-directed behavior.

**Methods:**

We recruited a sample of *n* = 30 participants with a psychotic disorder and at least mild negative symptoms and *n* = 30 healthy controls. We used the Balloon Effort Task, an effort-based decision-making paradigm, to assess amotivation on a behavioral level. We manipulated the salience of rewards in the paradigm by highlighting the monetary rewards in half of the trials.

**Results:**

Total effort expenditure did not differ between participants with and without psychotic disorders, but participants with psychotic disorders showed a significantly reduced effort allocation to the level of rewards. The salience of rewards manipulation significantly increased effort expenditure both in participants with psychotic disorders and in the healthy controls, but had no impact on effort allocation.

**Conclusions:**

Increasing the salience of reward cues promotes goal-directed behavior. This opens up new possibilities for interventions addressing amotivation in individuals with negative symptoms by facilitating the perception of reward cues.

**Supplementary Information:**

The online version contains supplementary material available at 10.1186/s12888-022-04274-7.

## Introduction

Amotivation is defined as reduced initiation and persistence of goal-directed behavior and is considered a core feature of negative symptoms in schizophrenia [1, 2]. Besides diminished expression, amotivation has been described as one of the primary dimensions of negative symptoms [3] and has been shown to predict reduced life satisfaction and levels of functioning [4–6]. Various psychological interventions have been developed to address amotivation, but their effects have not been satisfactory so far [7–9]. To develop more efficacious interventions, a more thorough understanding of the mechanisms that impede goal-directed behavior in individuals with psychotic disorders and negative symptoms is needed.

Several authors have suggested that goal-directed behavior could partly result from of an effort-cost computation that weighs the effort of various behaviors against the potential reward [10–13]. Results of studies using behavioral paradigms suggest that amotivation in the context of psychotic disorders could be caused by deviances in these effort-cost computations aside other factors [14–16]. Effort-cost computations have frequently been examined using so called effort-based decision-making paradigms in which participants choose between easy and a hard alternatives of performing a physically or cognitively demanding task (high vs. low effort). A monetary reward is offered upon completion of a trial, depending on the choice made: the easy choices are rewarded with a small sum while the hard choices are rewarded with variable higher sums (high vs. low reward). In eight studies reviewed by Green et al., individuals with psychotic disorders chose the hard alternative less frequently than healthy controls over the course of all trials (which has been referred to as total effort expenditure [17, 18]) and showed less adjustment of their choices to increasing levels of the reward across trials (which has been referred to as effort allocation [17, 18] and closely resembles the concept of reward sensitivity) [19]. Several researchers who have investigated effort-based decision making in individuals with psychotic disorders found reduced total effort expenditure and effort allocation in these paradigms to correlate with self−/observer-reported and observer-rated amotivation [20, 21]. Underpinning the relevance of deficits in effort-based decision making for real-life functioning in individuals with negative symptoms, studies using Ecological Momentary Assessment could demonstrate that the behavior in these paradigms predicts goal-directed behavior in daily life better than retrospective assessment methods for amotivation [22, 23]. However, it is unclear what mechanism underlies deviant effort-cost computations in these individuals.

The extent to which effort and reward enter the effort-cost computation depends on the individual’s perception of each of these two factors. In particular, an extenuated perception of the reward would result in a biased effort-cost computation that lacks the weight of the potential reward, which would then lead to a reduction in goal-directed behavior. Based on these considerations, several authors [20, 21] have suggested that different choice patterns between participants with psychotic disorders and healthy controls in effort-based decision-making paradigms may be due to deficits in their perception of reward cues. A reduced sensitivity to the expected reward due to an underlying perceptual deficit may explain why studies tend to report differences in effort allocation rather than in total effort expenditure [13, 24, 25]. It might also be a reason why a study by Bergé could not explain the reduced total effort expenditure in individuals with psychotic disorders and pronounced negative symptoms with cognitive deficits, such as reward representation and reward value [26]. We conclude that goal-directed behavior might be hampered by other stimulus-related processes, such as by an impaired perception of the reward cues. This hypothesis is also underpinned by functional magnetic resonance imaging studies suggesting that amotivation is associated with difficulties in prioritizing reward cues in the perception of environmental stimuli [27, 28].

If this interpretation holds true, then increasing the salience of reward cues should facilitate their prioritization and thereby increase goal-directed behavior. In support of this, Neumann et al. reported an association between performance in an effort-based decision-making paradigm and the self-reported prioritization of reward cues assessed with the Aberrant Salience Inventory [29] in a sample with schizophrenia patients [30]. However, the associations were no longer significant after correction for multiple testing and the cross-sectional design does not answer the question of causality. Hence, an experimental approach that manipulates the salience of reward cues to test the effect this has on goal-directed behavior seems a suitable way of moving the field forward.

### The present study

To this aim, we investigated the impact of the salience of reward cues as a factor potentially influencing effort-cost-computations underlying amotivation in individuals with negative symptoms in the context of a psychotic disorder. We manipulated the salience of reward cues in an effort-based decision-making paradigm, which we have previously found to enhance goal-directed behavior in a community sample [31], to test the impact of salience on total effort expenditure and effort allocation in individuals with psychotic disorders. First, in replication of previous research [24, 32], we hypothesized that individuals with psychotic disorders would less frequently select the hard alternative in the paradigm (main effect of group) and would show a reduced allocation of their effort to the level and probability of the reward as compared to healthy individuals (interaction effects of group with level and probability of the reward). Second, we hypothesized that in trials with high salience of monetary rewards participants would more frequently select the hard alternative (main effect of salience) and would show a stronger allocation of their effort to the level and probability of the reward (interaction effects of salience with level and probability of the reward) irrespective of group.

## Method

### Design, participants and procedure

We used a mixed design with two groups (psychosis and control) and a within-subject manipulation of reward salience.

An a priori power analysis for a medium between-subject and small within-subject and interaction effects based on α = .05 and β = .90 suggested a total sample size of 60 subjects. We therefore recruited a sample of *n* = 30 individuals with a current psychotic disorder with at least mild negative symptoms (psychosis group) and *n* = 30 healthy participants (control group) using bulletins in various public places such as stations, supermarkets, public placement services and the online employment platform *Stellenwerk* of the Universität Hamburg. Inclusion criteria for both groups were (1) age 18–65 years, (2) sufficient German skills, (3) no current substance intoxication, (4) no dementia/brain disease and (5) no considerable use of gambling machines in the past or present due to possible similarities with the effort-based decision-making paradigm which could confound the effect of our salience manipulation. Additional inclusion criteria for the psychosis group were (a) the current diagnosis of a psychotic disorder as indicated by the Structured Clinical Interview for DSM-5 (SCID-5-CV) [33] and (b) scoring ≥2 on at least either anhedonia or avolition in the Brief Negative Symptom Scale (BNSS) [34]. Additional inclusion criteria for the control group were (a) no current diagnosis of a mental disorder as indicated by the SCID-5-CV and (b) no first-grade relatives with a past/present psychotic disorder.

Upon written informed consent, participants began with a structured interview on demographic and clinical variables and the SCID-5-CV and participants of the psychosis group continued with the BNSS. Subsequently, participants of both groups completed an effort-based decision-making task and proceeded with questionnaires on amotivation and related constructs. Finally, participants received monetary compensation consisting of 10.00€ per hour and the monetary reward from the task which ranged from 3.20€ to 5.70€ depending on their choices.

### Measures

Negative symptoms in the psychosis group were assessed with the observer-rated BNSS (German version) [34, 35]. The original BNSS is a semi-structured interview with 13 items assessing anhedonia, distress, asociality, avolotion, blunted affect and alogia which are rated on a 6-point scale from 0 (normal) to 5 (severe). Following a two factor structure of negative symptomatology [3], it consists of the two subscales, motivation/pleasure and diminished expression. The psychometric properties of the original version [34] and the German version are very good (Cronbach’s α = 0.92) [35].

In addition, amotivation was assessed with the Motivation and Pleasure Scale – Self-Report (MAP-SR; German version) [36, 37]. The MAP-SR is a self-report scale that includes 15 items in self-report format derived from the Clinical Assessment Interview for Negative Symptoms motivation and pleasure scale [38]. It assesses motivation and consummatory pleasure as well as anticipatory pleasure in the following domains: activities with family, partner or friends, recreational activities and work. Items are rated on a 5-point scale with higher scores reflecting less motivation. The validity and reliability of the original MAP-SR [36] and the German version are very good (Cronbach’s α = 0.88) [37].

Apathy was assessed with the Apathy Evaluation Scale –Self-Report (AES-S; German version) [39, 40]. This self-report scale consists of 18 items that are rated on a 4-point scale with higher scores reflecting less motivation (e.g. “Getting things started on my own is important to me”). The psychometric properties of the AES-S are also very good (Cronbach’s α = 0.91) [39, 40].

Depression was assessed with the Center for Epidemiological Studies Depression Scale [41] (German version: Allgemeine Depressionsskala; ADS) [42]. The ADS is a self-report scale with 20 items to assess symptoms of depression including emotional, motivational, cognitive, somatic and interactional impairments over the last week and has very good psychometric properties (Cronbach’s α = 0.89) [42].

### Effort-based decision-making task

Motivation in terms of total effort expenditure and effort allocation for receiving a reward was assessed with the Balloon Effort Task [24]. In this effort-based decision-making paradigm participants are shown two balloons on a computer screen. The balloon on the left hand side is labelled “easy”, the balloon on the right hand side “hard”. Participants are asked to choose whether they inflate either the “easy” or the “hard” balloon by repeated button presses until the balloon bursts. Inflation of the “easy” balloon requires 20 button presses, whilst the “hard” one requires 100 button presses. In each trial, the levels of the monetary rewards that can be achieved in the respective trial and the probability to receive it are displayed on the screen. The completion of the easy balloon is rewarded with 1€ in each trial, whereas the level of the reward for the completion of the hard balloon varies across trials from 3 to 7€. The probability to receive the reward is 100% in one half and 50% in the other half of a total of 30 trials that follow a pseudo-randomized order. Participants were told that they will receive 5% of the total earnings from all trials and were instructed on the procedure prior to beginning the task, which does not include training trials. The original version of the Balloon Effort Task was found to be one of the most promising effort-based decision-making paradigms in the context of psychotic disorders in a review by Horan et al., both in terms of in its psychometric properties and in terms of participants’ acceptance [20].

For the purpose of this study, we manipulated the salience of the reward in half of the trials. In salient trials, the indications of the monetary rewards for both of the balloons were presented in bright yellow (rather than dark red as in the original paradigm), were twice the size of the original paradigm, were programmed to blink and were introduced with a short tune. Salient trials appeared in a randomized order and the levels of the rewards as well as the probabilities of rewards were balanced across salient and normal trials. Stimuli were presented in *E-Prime* Version 2.0 on a 22 in. computer screen. A visualization of trials is included in the Additional file 1. We used the percentages of choices for the hard balloon (hard choices) as outcomes.

### Data analysis

Statistical analyses were carried out using SPSS 26. We first conducted t- and *X*^2^ - tests to test for baseline group differences in demographic and clinical variables. To ensure comparability with previous studies on the Balloon Effort Task and other effort-based decision-making paradigms [13, 24, 25], we chose to conduct an ANOVA to test the group differences and manipulation effects on the total effort expenditure and on the allocation to the level and the probability of the reward. We therefore conducted a 2 X 5 X 2 X 2 mixed ANOVA with hard choices as the criterion. There were three within-subject-factors, salience (normal/salient), reward level (3€/4€/5€/6€/7€) and reward probability (50%/100%), and group as the between-subject-factor. We explored correlations between measures for amotivation and related constructs (overall hard choices in the Balloon Effort Task, BNSS, MAP-SR, AES-S, ADS) for the total sample. All tests were performed with α = .05. For the purpose of our replication hypotheses, we repeated all analyses after excluding the subsample of inflexible responders (i.e. participants who always chose the hard task) as has been done in several previous studies on the Balloon Effort Task [20, 32]. Findings on this subsample are only reported when they diverge from the full sample.

## Results

### Sample characteristics and preliminary analysis

The sample consisted of 30 outpatients with a diagnosis of either schizophrenia (*n* = 14), schizoaffective disorder (*n* = 11) or psychotic disorder not otherwise specified (*n* = 5) in the psychosis group and 30 healthy participants in the control group. Descriptive data are shown in Table 1. The psychosis and the control group did not significantly differ in age, gender or level of education. Participants of the control group had a significantly higher income and were significantly more often employed in a full-time job than participants of the psychosis group. Due to technical problems, data from the Balloon Effort Task was missing in two cases in the psychosis and in one in the control group. There were four inflexible responders, three in the psychosis and one in the control group.

**Table 1:** Demographic and clinical variables for participants with psychotic disorders and motivational negative symptoms and healthy controls

	Psychosis (*n* = 30)	Healthy (*n* = 30)	*t / X*^2^	*p*
Age in years (SD)	38.60 (9.27)	39.63 (14.02)	0.337	.738
Gender (male/female)	21/9	18/12	0.803	.425
Level of education	0/13/17	0/11/19	0.278	.598
Employment status	5/10/15	16/10/4	12.130	.002**
Individual net income in €	1082	1871	3.465	.001**
Years of psychotic disorder (SD)	12.69 (8.16)	–	–	–
Antipsychotic medication (yes/no)	20 / 10	–	–	–
BNSS total (SD)	22.97 (14.00)	–	–	–
BNSS motivation (SD)	14.40 (8.60)	–	–	–
MAP-SR total (SD)	24.70 (9.99)	16.83 (5.37)	3.798	<.001***
MAP-SR motivation (SD)	10.87 (5.37)	7.97 (3.25)	2.531	.015*
AES-S total (SD)	17.83 (8.18)	10.93 (6.28)	3.666	.001**
ADS total (SD)	20.90 (9.17)	9.70 (5.52)	5.732	<.001***

*Level of education* no degree/secondary education/higher education, *Employment status* full time/part time/unemployed, *BNSS* Brief Negative Symptom Scale, *MAP-SR* Motivation and Pleasure Scale, self-report, *AES* Apathy Evaluation Scale, self-report, *ADS* Allgemeine Depressionsskala [General Depression Scale]

*p ≤ .05 **p ≤ .01 ***p ≤ .001

### Effect of group and salience of rewards on total effort expenditure and allocation in the balloon effort task

**Fig. 1:**
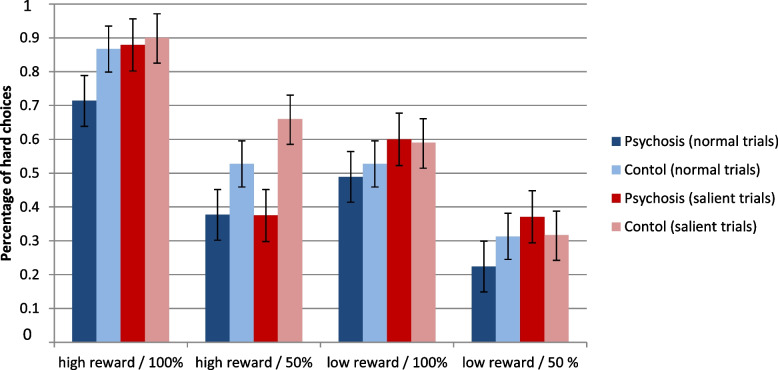
Performance in the effort-based decision-making paradigm. Percentages of choices for the hard effort alternative in the Balloon Effort Task for high (6€,7€) / low (3€, 4€) reward level and for 100% / 50% reward probability in normal trials and trials with salient reward for the psychosis and the control group with standard errors

**Fig. 2:**
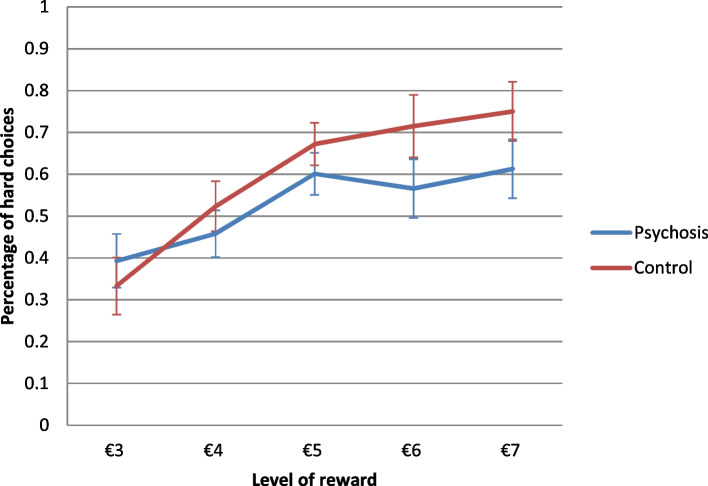
Interaction effect of reward and group on performance in the effort-based decision-making paradigm. Percentages of choices for the hard effort alternative in the Balloon Effort Task for the five levels of reward collapsed across the factors probability and salience. Separate lines for the psychosis and the control group with standard errors for each group

Percentages of hard choices in salient and normal trials for both groups are displayed in Fig. 1. The ANOVA of hard choices as outcome with the independent variables salience, level and probability of the reward, and group can be seen in Table 2. There were significant main effects for level and probability of the reward and a significant interaction effect of the two factors indicating that participants chose the hard alternative more often in trials with high reward and in trials with high probability and that the combination of both factors increased hard choices even more. Percentages of hard choices for the five levels of reward for both groups are displayed in Fig. 2. In support of the second hypothesis, there was a significant main effect of salience with a medium to large effect size, indicating that participants chose the hard alternative more often in salient as compared to normal trials. In contrast to the second hypothesis, there were no significant interaction effects of salience with any of the other factors. In contrast to the first hypothesis, there was no significant main effect of group. In support of the first hypothesis, there was a significant interaction effect of group with the level of the reward with a medium effect size, indicating that the control group showed a stronger increase in hard choices with higher rewards than the psychosis group. No other interaction effects of group with any of the remaining factors were significant. All post-hoc t-tests were non-significant after Bonferroni-correction.

**Table 2:** Results of ANOVA with Balloon Effort Task hard choices as the criterion

Predictor	*MSE*	*F*	*df*	*p*	*η*^2^
Reward	0.19^a^	31.71	2.98,163.91	<.001***	.366
Probability	0.35	84.22	1,55	<.001***	.605
Salience	0.93	8.80	1,55	.004**	.138
Group	1.25	1.51	1,55	.225	.027
Reward x probability	0.11	7.56	4220	<.001***	.121
Reward x salience	0.09	1.93	4220	.107	.034
Reward x group	0.19^a^	3.18	2.98,55	.026*	.055
Probability x salience	0.10	0.06	1,55	.802	.001
Probability x group	0.52	0.78	1,55	.380	.014
Salience x group	0.93	0.00	1,55	.997	.000
Reward x probability x salience	0.10^a^	0.91	3.41,178.38	.446	.016
Reward x probability x group	0.13	1.59	4,55	.177	.028
Reward x salience x group	0.09	0.43	4,55	.787	.008
Probability x salience x group	0.10	1.55	1,55	.218	.027
Reward x probability x salience x group	0.10^a^	1.85	3.41,55	.132	.032

*MSE* Mean Squared Error

^a^Greenhouse–Geisser correction for violations of sphericity. *p ≤ .05 **p ≤ .01 ***p < .001

In support of the first hypothesis, in the additional analysis of the subsample after the exclusion of inflexible responders revealed a significant main effect of group, *F*(1,51) = 4.19, *p* = .046, partial η^2^ = .076 indicating that participants of the control group made a hard choice more frequently than participants of the psychosis group. The pattern of significance of all other main and interaction effects did not differ from the results of the full sample.

### Additional analyses

We repeated the mixed ANOVA analysis of hard choices as outcome with the independent variables salience, level and probability of the reward, and group but with only two levels of reward (high = 6 and 7 € / low = 3 and 4 €). The pattern of results of this 2 X 2 X 2 X 2 ANOVA did not differ from the ANOVA reported for five levels of reward apart from a now significant interaction effect of the four factors, *F*(1,55) = 6.77, *p* = .012, partial η^2^ = .110, indicating that the differences in hard choices between the psychosis and the control group were smaller in salient trials with both high reward and probability. All post-hoc t-tests were non-significant after Bonferroni-correction.

The correlations between questionnaires and hard choices in the Balloon Effort Task for the total sample are presented in Table 3. There were no significant correlations between overall hard choices or hard choices in normal (non-salient) trials and any of the questionnaires. There were significant correlations between hard choices in salient trials and amotivation as well as apathy. In an analysis of both groups separately, there were no significant correlations between questionnaires and hard choices in the Balloon Effort Task. In the psychosis group there were no significant correlations between the BNSS and overall hard choices or hard choices in normal or salient trials.

**Table 3:** Correlations between questionnaires and Balloon Effort Task hard choices for the total sample *N* = 60

	M (SD)	1	2	3	4	5	6
1. MAP-SR	20.8 (8.9)	–	–	–	–	–	–
2. MAP-SR motivation	9.4 (4.6)	.90***	–	–	–	–	–
3. AES-S	14.4 (8.0)	.63***	.54***	–	–	–	–
4. ADS	15.3 (9.4)	.59***	.49***	.52***	–	–	–
5. Balloon total^a^	.563 (.25)	−.26	−.24	−.25	−.12	–	–
6. Balloon salient^a^	.587 (.25)	−.31*	−.29*	−.27*	−.12	.96***	–
7. Balloon normal^a^	.546 (.27)	−.14	−.18	−.23	−.12	.97***	.86***

*MAP-SR* Motivation and Pleasure Scale, self-report, *AES* Apathy Evaluation Scale, self-report, *ADS* Allgemeine Depressionsskala [General Depression Scale], *Balloon total* Balloon Effort Task, percentage of hard choices in all trials, *Balloon salient* Balloon Effort Task, percentage of hard choices in trials with salient reward, *Balloon normal* Balloon Effort Task, percentage of hard choices in normal trials

*p ≤ .05 ** *p* ≤ .01 ***p < .001

^a^Variables with *n* = 57 due to missing values

There were no significant associations between hard choices in the Balloon Effort Task and age, level of education, income, years of the psychotic disorder or antipsychotic medication. In a 2 X 5 X 2 X 2 mixed ANOVA with the between-subject-factor gender and the within-subject-factors salience, reward and probability and hard choices as the criterion, there was a significant effect of gender, *F*(1,55) = 7.24, *p* = .012, partial η^2^ = .110, indicating that women made a hard choice more frequently. There was a significant interaction effect of gender with probability, *F*(1,55) = 13.51, *p* = .001, partial η^2^ = .197, indicating that women made hard choices more frequently than men in trials with low rewards but not in trials with high rewards. No other interaction effects with gender were significant.

## Discussion

We tested the effect of salience of reward incentives on overall effort expenditure for rewards and effort allocation in an effort-based decision-making paradigm in individuals with motivational negative symptoms in the context of psychotic disorders and healthy controls. In line with our hypotheses, participants of both groups chose the high-effort alternative in the Balloon Effort Task significantly more often in salient as compared to normal trials with a medium to large effect size. There were no significant interaction effects between the level and the probability of the reward with salience indicating that the salience of rewards had no significant impact on effort allocation. We found no significant difference between the psychosis and the control group in overall effort expenditure in the total sample. After excluding participants with an inflexible response pattern, we found the expected effect with a medium effect size, of healthy controls choosing the hard alternative more often. In terms of effort allocation, we found that participants’ choice behavior in the control group was more sensitive to the amount of reward. Increasing the salience of reward cues increased hard choices in both individuals with psychotic disorders and healthy controls.

The finding that increasing the salience of reward cues increased approach behavior offers promising new avenues for clinical interventions aimed at improving approach behavior in individuals with negative symptoms. To make rewards more salient in every-day life, therapists could, for example, support patients in improving their perception of the environment and in better recognizing reward incentives. A pilot program addressing apathy in patients with schizophrenia that aimed to increase the incentive values through the use of imagination techniques and anticipatory pleasure exercises has already shown promising effects [43]. We therefore propose to further explore the possibilities of addressing reward salience in interventions for negative symptoms.

In contrast to overall effort expenditure, we did not find reliable evidence for salience to also increase effort allocation. One explanation might be that only the reward values were made salient but not the probabilities. This seems plausible considering that the effect size of the salience-reward-interaction was greater than the salience-probability-interaction and might have reached significance in a larger sample. Our more robust analysis with combined reward levels revealed that under certain circumstances (e.g., when potential reward is high and certain) salience affected effort allocation slightly more in the psychosis group. This could indicate that highlighting the salience helped the psychosis sample to increase their sensitivity to the amount of rewards.

We did not find a main effect of group on overall hard choices in the total sample. Nonetheless, we were able to replicate the finding of a significant main effect by Reddy et al. after the exclusion of inflexible responders as suggested by the authors [32]. Unlike Gold et al. who reported an interaction effect of reward probability with group [24], we found a significant interaction effect of group with the level of the reward. Compared to other effort-based decision-making tasks, the Balloon Effort Task has not been as widely used in samples with psychotic disorders. It is therefore too early to draw final conclusions on its ability to assess potential deficits in total effort expenditure or effort allocation and their association with motivational negative symptoms.

Finally, there were only few significant correlations between the questionnaires tapping into amotivation, apathy and depression and the overall hard choices in the effort-based decision-making paradigm. This pattern of findings is in line with previous studies on the associations between performance in effort-based decision-making paradigms with self-reported and observer-rated amotivation: A meta-analysis [21] suggested a significant yet small correlation between these two measurement methods for amotivation, however, many studies failed to find any association [ 24, 32]. Discrepancies between the two methods might be explained by factors such as differences in time-spans of assessment or by the specificity of the behavior required by the tasks in contrast to the questionnaires that aim to capture a wide range of potential behaviors.

A limitation of this study is that due to the Covid-19 pandemic we were not able to include inpatients. This might explain why patients in our sample had lower scores on the BNSS and its’ motivation subscale as compared to the German validation sample [35]. Thus, our sample might be biased towards the more functional. In addition, although the groups were matched in terms of age, gender and level of education, individuals of the psychosis group had a significantly lower income which could have led to a higher motivation for them to earn money from their participation in the Balloon Effort Task as compared to participants in the control group. Moreover, both, the samples’ characteristics and their size may have limited the ability to find small between group effects in task performance. Another limitation is that the addition of a third within-subject factor in the Balloon Effort Tasks, the salience of rewards, resulted in a combination of each of the factor levels in one or two trials only. To assure the validated properties of the paradigm and the comparability to other studies, we decided not to add more trials. As the three factors were completely counterbalanced, this had no impact on the ability to find main effects. Nevertheless, it may have limited the ability to find the intended interaction effects. By choosing the Balloon Effort Task we opted for a paradigm with good psychometric properties and participants’ acceptance, however it needs mentioning that the overall effort expenditure in this task was higher than has been reported for other effort-based decision-making paradigms. This might have limited the power to find effects of the independent variables. Further, our salience manipulation might have increased arousal levels, which have been shown to cause their own effects on the processing of visual stimuli and motivational processes [44] and could therefore have added to the effects on both total effort expenditure and allocation. We therefore suggest to control for arousal levels and explore effects on approach behavior in future research.

To conclude, we found evidence for the assumption that increasing the salience of rewards has the potential to significantly improve overall effort expenditure in the Balloon Effort Task in individuals with and without psychotic disorders. Whether and how this effect can be translated to improve goal-directed behavior in real-life settings and how it can be incorporated into psychological interventions for amotivation should be explored in future research.

## Supplementary Information

**Additional file 1.**

## Data Availability

The materials for the experiments reported and the datasets analyzed in the current study are available from the corresponding author on reasonable request.
