# Supplementary material for: The effect of salience of rewards on effort-based decision making in psychotic disorders
Source: BMC Psychiatry. 2022 Oct 13;22:643. doi: 10.1186/s12888-022-04274-7 (PMC9559256; doi:10.1186/s12888-022-04274-7)
Supplement: Supplementary file 1 — Additional file 1. [file 12888_2022_4274_MOESM1_ESM.docx]

**Additional file 1**
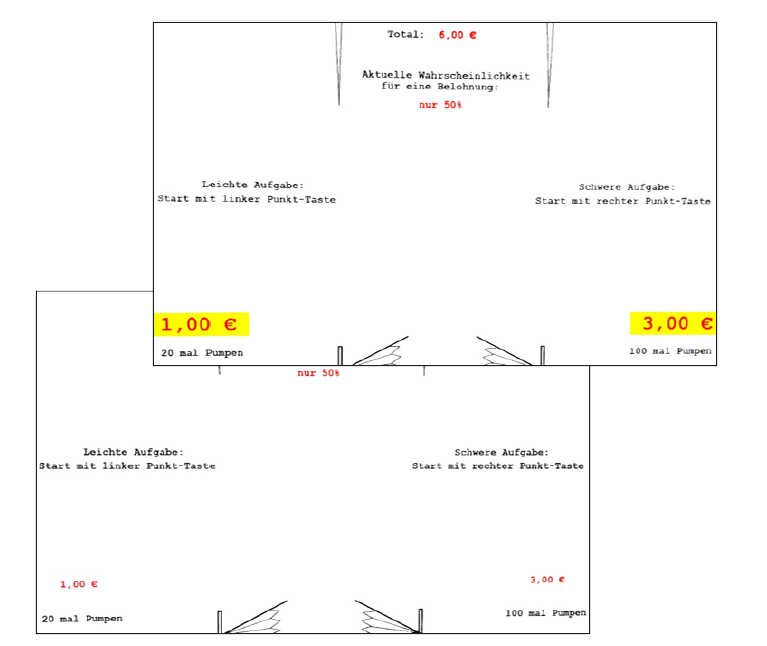


Decision phase in the Balloon Effort Task in salient and normal trials.


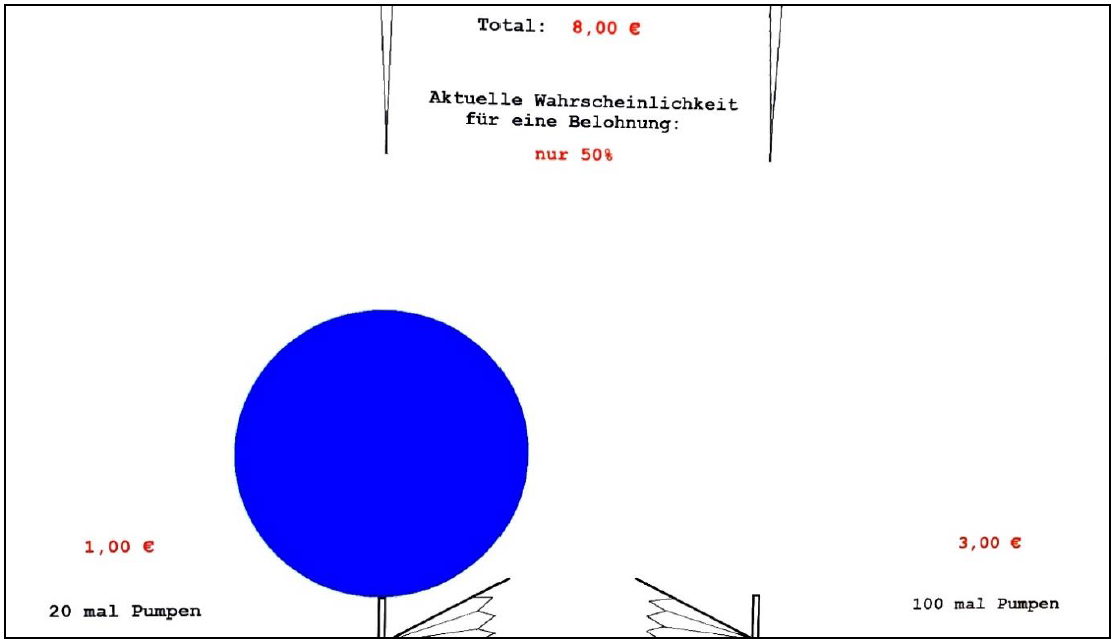


Balloon pumping phase.
